# Huoxue Jiedu Huayu Recipe Ameliorates Mesangial Cell Pyroptosis in Contralateral Kidney of UUO Rats

**DOI:** 10.1155/2020/2530431

**Published:** 2020-12-29

**Authors:** Yuxuan Zhang, Juan Hao, Xuelian Ma, Qiyue Zhao, Xiaomeng Gao, Xiangting Wang, Qingyou Xu

**Affiliations:** ^1^Graduate School, Hebei University of Chinese Medicine, Shijiazhuang, China; ^2^Hebei Key Laboratory of Integrative Medicine on Liver-Kidney Patterns, Hebei University of Chinese Medicine, Shijiazhuang, China; ^3^Department of Internal Medicine, Hebei University of Chinese Medicine, Shijiazhuang, China

## Abstract

**Objectives:**

To observe the effects of the Huoxue Jiedu Huayu Recipe (HJHR) on pyroptosis of glomerular mesangial cells in the contralateral unobstructed kidney (CK) of unilateral ureteral obstruction (UUO) rats.

**Methods:**

Sprague-Dawley rats were randomly divided into 4 groups: sham group, UUO group (10 days of left ureter ligation), UUO treated with eplerenone (EPL) (UUO + EPL) group, and UUO treated with HJHR (UUO + HJHR) group. The CKs of all rats were collected for studies.

**Results:**

Cell pyroptosis and macrophage infiltration was found in contralateral glomeruli, and nucleotide-binding oligomerization domain-like pyrin domain containing protein 3 (NLRP3) and interleukin (IL)-1*β* expression was upregulated in the CK of UUO rats. All of these changes were inhibited by HJHR and eplerenone. To determine how aldosterone (Aldo) activated the mineralocorticoid receptor (MR) and then induced mesangial cell pyroptosis with NLRP3-caspase-1-IL-1*β* pathway, human mesangial cells (HMCs) were treated with HJHR and eplerenone, which were examined to detect the expression of NLRP3 inflammasome-associated proteins following treatment with Aldo.

**Conclusion:**

These results suggest that HJHR and eplerenone suppressed HMC pyroptosis via the MR/NLRP3 pathway.

## 1. Introduction

In the clinic, obstructive nephropathy has emerged as a major cause of end-stage renal diseases (ESRDs), especially in the countryside of China [1, 2]. In fact, the renal lesions of obstructive uropathy not only persist but also progress long after unilateral ureteral obstruction (UUO) is relieved in both adults and newborns. Our previous studies have demonstrated that activated mineralocorticoid receptor (MR) in the contralateral unobstructed kidney (CK) of UUO plays an important role in the progression of renal interstitial fibrosis with the serum- and glucocorticoid-inducible protein kinase-1 (SGK-1) pathway [3]. Glomerular inflammation preceding interstitial fibrosis is a major process involved in the progression of glomerulosclerosis [4], and partial renal function loss on the CK accelerates progression of renal failure. Mesangial cells, glomerular endothelium, and epithelial cells, as well as podocyte apoptosis and pyroptosis, are closely related to glomerulosclerosis. Increasing evidence demonstrated that inflammation is attributable to the development and progression of kidney injury [4, 5]. It also has been shown that nucleotide-binding oligomerization domain-like pyrin domain containing protein 3 (NLRP3) inflammasome contributed to the pathogenesis of renal injuries resulting from UUO [6]. Therefore, in this study, the contralateral glomerulus was used as the research object to explore the mechanism of CK aggravating the progression of chronic renal failure. We demonstrated that UUO-induced contralateral glomerular injury was characterized by mesangial cell pyroptosis. In the present study, we observed that the UUO-induced contralateral glomerular injury was prevented by treatment with Huoxue Jiedu Huayu Recipe (HJHR) and eplerenone. We also found that glomerular mesangial injury was associated with the activation of MR/NLRP3 in CK. These results may further be the knowledge on the pathophysiology of obstructive nephropathy and lead to novel treatment options for glomerular injury using HJHR and eplerenone.

## 2. Methods

### 2.1. Drug Preparation

Huoxue Jiedu Huayu Recipe was composed of Shenghuangqi (Radix Astragali seu Hedysari) 20 g, Dilong (*Lumbricus*) 10 g, Biejia (Carapax Trionycis) 10 g, Chishao (Radix Paeoniae Rubra) 10 g, Huangqin (Radix Scutellariae) 10 g, and crude drug at 1 g/mL liquid. Those herbals were purchased from Shijiazhuang Lerentang Pharmaceutical Co., Ltd., and the herbal drugs were identified and decocted in water.

### 2.2. Animal Studies

Sprague-Dawley rats were randomly divided into 4 groups: the sham group, the UUO group, eplerenone group, and HJHR group. The rats in the UUO, eplerenone, and HJHR groups underwent unilateral ureteral ligation through a midline abdominal incision under sterile conditions as routinely carried out in our lab [7]. In the sham group, the left ureter was exposed but not ligated. For the eplerenone group, the rats were orally administered mixed eplerenone with diet (1.25 g/kg) after UUO [3]. The HJHR group was intragastrically administrated with 11.7 g/kg HJHR every day. However, rats in the UUO and sham groups were intragastrically administrated with the same volume of physiological saline. Kidneys were harvested at day 10. Sprague-Dawley rats (male, 200 ± 10 g) were purchased from the Hebei Province Experimental Animal Centre (SCXK 2013-1-003; *n* = 40). All experimental procedures were performed in accordance with the Provision and General Recommendation of the Chinese Experimental Animal Administration Legislation.

### 2.3. Transferase-Mediated dUTP Nick-End Labelling (TUNEL) Studies

Pyroptosis in cultured cells or CK tissue sections was identified using the TUNEL assay (Roche, no. 35181600). Investigation of positive cells was performed using terminal deoxynucleotidyl TUNEL with a DeadEnd Fluorometric TUNEL System, according to the manufacturer's instructions (Roche). For quantification, 10 fields were randomly selected from each tissue section or cell, and the number of TUNEL-positive cells was counted per millimetre.

### 2.4. Histological Analysis

For histologic analysis, 4 *μ*m sections of paraformaldehyde (4%, neutrally buffered)-fixed paraffin-embedded rat kidneys were stained with haematoxylin and eosin (H&E) and Masson to verify cell damage and collagen deposition in the CK.

### 2.5. Immunohistochemistry

After fixation and paraffin embedding, 4 *μ*m sections were deparaffinised with xylene and rehydrated by passing through graded ethanol. Endogenous peroxidase activity was inactivated with 3% hydrogen peroxide in 100% methanol for 20 min after antigen retrieval with heat in 10 mM of citrate buffer. Next, 10% normal goat serum in phosphate-buffered saline (PBS) was added to the sections for 30 min at 37°C to block nonspecific antibody binding. Then, sections were incubated with the NR3C2 (Abcam, no. GR195919-1), MCP-1 (Abcam, no. GR1151-61), and nuclear factor *κB* (NF-*κB*) (Abcam, no. GR3236344-1) primary antibodies overnight at 4°C in PBS. After rinsing in PBS, sections were incubated with biotinylated secondary antibody and horseradish peroxidase-conjugated streptavidin. Labels were visualised with diaminobenzidine (DAB) to produce a brown colour as positive expression, and sections were counterstained with haematoxylin.

### 2.6. Immunofluorescence Assay

Expressions of F4/80 (Abcam, no. GR207473-22), NLRP3 (Novus Biologicals, no. 08063965C-05), and serum- and glucocorticoid-inducible protein kinase-1 (SGK-1) (Affinty, no. 19U71) in renal tissues were detected using immunofluorescence assays. Frozen renal tissue sections were incubated with primary antibodies in phosphate buffer saline (PBS) overnight at 4°C. After washing, renal tissues were incubated with TRITC/FITC (tetramethylrhodamine isothiocyanate)/(fluorescein isothiocyanate)-labelled secondary antibodies for 1 h at 37°C and photographed with confocal microscopy (Leica, SP8).

Cells cultured in 6-well chamber slides were fixed with 4% paraformaldehyde for 1 h at 4°C and permeabilised with 0.1% Triton *X*-100 for 10 min at 37°C. After washing, cells were incubated with antibodies against MR (Abcam, no. GR195919-1), NF-*κB* (Abcam, no. GR3236344-1), and NLRP3 (Novus Biologicals, no. 08063965C-05) overnight at 4°C. Then, cells were incubated with the TRITC/FITC-labelled secondary antibody (1 : 200) for 1 h at 37°C. After washing with PBS, cell nuclei were stained with 4, 6′-diamidino-2-phenylindole (DAPI) for 5 min. Images were obtained using EVOS.

### 2.7. Protein Extraction and Western Blotting

Cells or kidney tissues were homogenised in a lysis buffer according to the standard procedures. 30∼60 *µ*g of protein from the whole cell preparation was denatured in boiling water for 15 min, separated on sodium dodecyl sulfate-polyacrylamide gel electrophoresis (SDS-PAGE) gel and transferred onto polyvinylidene difluoride (PVDF) membranes. Immunoblotting was performed with primary antibodies against NLRP3 (1 : 1,000), caspase-1 (1 : 500), and IL-1*β* (1 : 1,000). Next, the membranes were incubated with fluorescein-conjugated secondary antibodies (room temperature, 1 h, 1 : 20,000). Protein levels were adjusted by glyceraldehyde-3-phosphate dehydrogenase (GAPDH).

### 2.8. Mesangial Cell Culture

Cultures of human mesangial cells were supported kindly by Professor Shiyonghong from Hebei Medical University. The cells were maintained in a special media supplemented with 1 g/L D-glucose DMEM (GIBCO), 5%–10% foetal calf serum (GIBCO), and 1% Pen-Strep (GIBCO). Cells in the third to seventh passages were used in all in vitro experiments. Depending on different experiments, HMCs were pretreated with a serum-free medium for 24 h and then stimulated with DMEM (control), Aldo (1 *μ*M), aldosterone with eplerenone (10 *μ*M), and aldosterone with serum containing the HJHR at indicated time points.

### 2.9. Statistical Analysis

Data are expressed as the means ± standard error of the mean (SEM). Statistical analysis was performed using GraphPad Prism 5 (GraphPad Software Inc., San Diego, CA, USA). The results were analysed by analysis of variance (ANOVA) with Tukey's post hoc test. *P* < 0.05 was considered statistically significant.

## 3. Results

Animals were subjected to UUO for 10 days, and CKs were harvested at that time. The animals were treated with HJHR as detailed in materials and methods. We examined several parameters of renal damage. All contralateral unobstructed kidneys were compared with sham rats. HMCs were stimulated with Aldo (1 *μ*M), Aldo with eplerenone (10 *μ*M), and Aldo with HJHR for 24 h, and then the cells used for study were collected.

### 3.1. UUO-Induced Inflammation in the Contralateral Glomeruli is Reversed by MR-Blocker and HJHR

**Figure 1:**
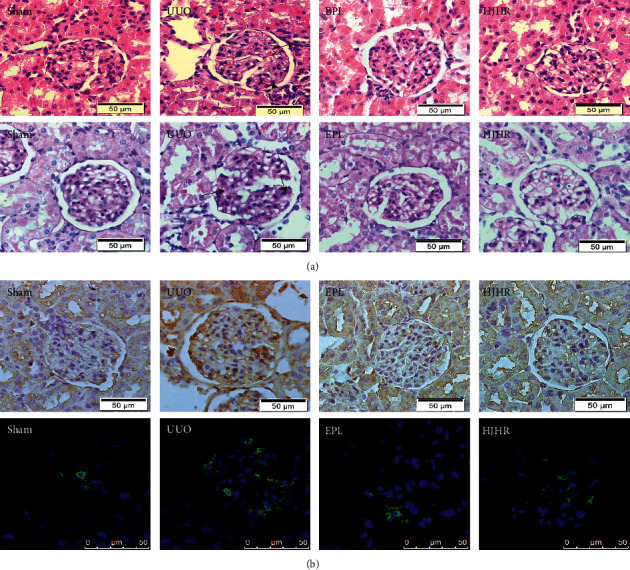
(a) HJHR alleviated mesangial cell injury after UUO. After 14 days of UUO, contralateral kidney sections were stained with Masson and haematoxylin and eosin (H&E). (b) The expression of MCP-1 and F4/80 in the contralateral glomeruli of the sham, UUO, and EPL rats. Both immunostains were increased in UUO rats and reduced by HJHR and eplerenone (scale bar indicates 50 *μ*m).

We first confirmed the inflammation in the contralateral glomeruli of the UUO model. Histomorphology was assessed by H&E and Masson staining. We also stained monocyte chemoattractantprotein-1 (MCP-1) and F4/80 to show the degree of inflammation. F4/80 is the marker of rat macrophages which have classically been recognised as an active player in progressive renal scarring. The results showed that there were no changes on glomeruli from the sham group, and the glomerular structures were well preserved with a few inflammatory cells, and little collagen deposition was observed in the contralateral glomeruli. There was a significant increase in macrophage infiltration into the CK compared with the sham after 10 days of UUO, and it was ameliorated in the eplerenone group and HJHR group (Figure 1).

### 3.2. HJHR and Eplerenone Ameliorate Glomerular Cell Injury Detected by TUNEL Assay

**Figure 2:**
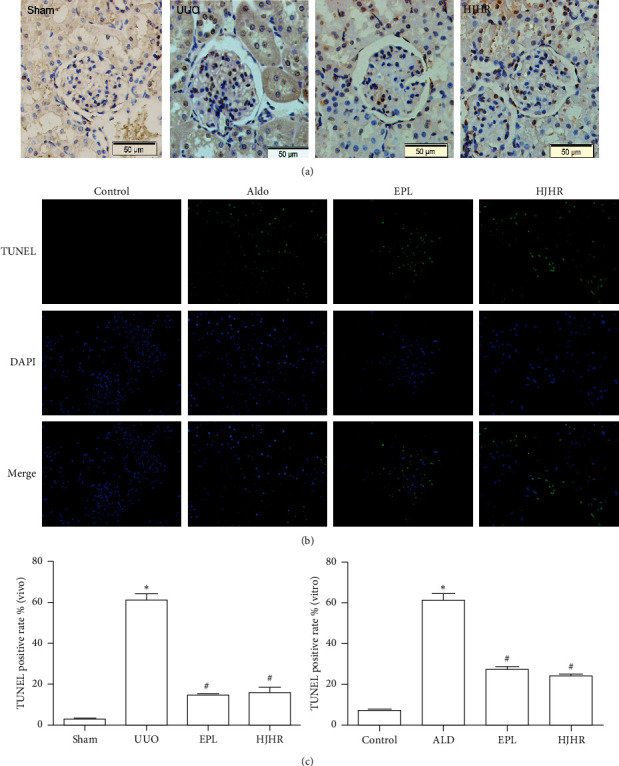
(a) Effect of HJHR ameliorates mesangial cell pyroptosis in the contralateral kidneys of UUO rats through TUNEL. The rats were randomly divided into 4 groups: the sham group, the UUO group, the UUO with eplerenone group (100 mg·kg^−1^·day−^1^), and UUO with HJHR group. Renal sections were evaluated for TUNEL-positive cells. Sham: representative photomicrographs of contralateral glomeruli. UUO: representative photomicrographs of glomeruli from UUO rats. EPL: representative photomicrographs of glomeruli from UUO with eplerenone-treated rats. HJHR: representative photomicrographs of glomeruli from UUO with HJHR-treated rats. Positive cells showed brown staining. (b) Effect of eplerenone ameliorates mesangial cell injury by TUNEL. Human mesangial cells were treated for 4 groups: control group; Aldo group (1 *μ*M Aldo); EPL group (10 *μ*M EPL with 1 *μ*M Aldo); HJHR group (HJHR with 1 *μ*M Aldo). Equal numbers of cells were incubated for 24 h. (c) Quantitative analysis of TUNEL-positive cells in vivo and vitro.

We investigated DNA damage by TUNEL staining, as shown in Figure 2(a) in the CK, 10 days of UUO resulted in a significant increase in TUNEL-positive cells compared with the sham glomerulus, and eplerenone and HJHR partially inhibited effects. In in vitro experiments, as shown in Figure 2(b), aldosterone promoted MC injury. However, this effect of aldosterone was inhibited by eplerenone and HJHR. In a parallel series of experiments, cells were treated under similar conditions.

### 3.3. HJHR and Eplerenone Blocked MR Activity

**Figure 3:**
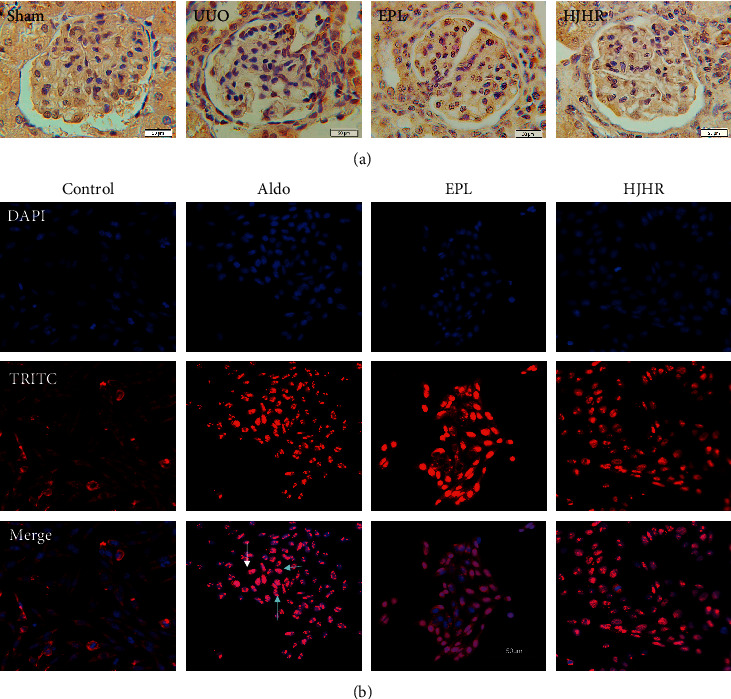
(a) Expression of mineralocorticoid receptor (MR). Rat contralateral glomerular sections were analysed by immunohistochemistry using antibodies against MR. (b) Human mesangial cells were treated for 24 h with vehicle or 1 *μ*M of aldosterone at 37°C. Cells were fixed and analysed by EVOS using antibodies against MR (red staining). Cells were also stained with DAPI (nucleus, blue staining).

MR plays key roles in the cell pyroptosis or apoptosis. Normally, the MR is inactive. Treatment with aldosterone resulted in translocation of MR to the nucleus. Recent studies have demonstrated that aldosterone contributes to the progression of renal injury via direct actions on mesangial cells, through the activation of locally expressed MR [8]. To determine whether MR is expressed in mesangial cells and involved in aldosterone-induced MC injury, we performed immunofluorescence on kidney sections and HMCs. In the sham group, MR is mainly expressed cytoplasm. The aldosterone group shows that MR is mainly expressed in the nucleus. However, it is inhibited by HJHR and eplerenone. These results indicate that the glomerular mesangium is a target for injuries induced by aldosterone via activation of MR. Huoxue Jiedu Huayu Recipe ameliorates mesangial cell pyroptosis by inhibiting  MR activation, as shown in Figure 3.

### 3.4. HJHR and Eplerenone Inhibited Aldo-Induced NLRP3 Expression

**Figure 4:**
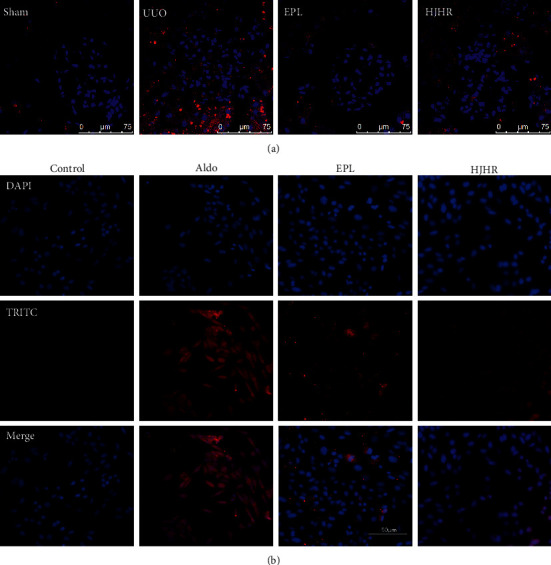
Expression of NLRP3. (a) Rat glomerular sections were analysed by immunofluorescence using antibodies against NLRP3 (red staining). Sections were also stained with DAPI (nucleus, blue staining). (b) Human mesangial cells were treated for 24 h with vehicle, 1 *μ*M aldosterone, 1 *μ*M Aldo with eplerenone (100 *μ*M), and 1 *μ*M Aldo with HJHR at 37°C.

A previous study has suggested activation of the NLRP3 inflammasome following 14 days of UUO [6]. Therefore, we investigated renal expression of NLRP3 during the development of renal injury in CK of UUO. As shown in Figure 4(a), the expression levels of NLRP3 in CK of UUO were increased compared with the sham group and significantly decreased in the HJHR group and eplerenone group as compared with the UUO group. To determine the role of NLRP3 in UUO-induced MC pyroptosis, equal numbers of cells were incubated in a medium containing either buffer, aldosterone (1 *μ*M), aldosterone with eplerenone (10 *μ*M), and aldosterone with HJHR for 24 h. Subsequently, cells were prepared for IF. In a parallel series of experiments, cells were treated under similar conditions.  Figure 4(b) shows the expression of NLRP3 in HMC of the four groups. As compared with the control group, the expression of NLRP3 in the UUO group was significantly increased. In the HJHR group and eplerenone group, the expression of NLRP3 was downregulated.

### 3.5. HJHR and Eplerenone Ameliorate Inflammatory Cytokine Expression

**Figure 5:**
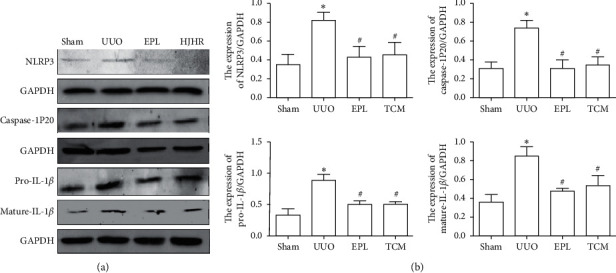
Inhibitory effect of HJHR on inflammation in the contralateral kidney of rats with UUO. (a) Western blot analysis of NLRP3, NLRP3, pro-caspase-1, pro-IL-1*β*, caspase-1-p20, and mature-IL-1*β* in the contralateral kidney. (b) Quantification of NLRP3/GAPDH, caspase-1/GAPDH, and IL-1*β*/GAPDH protein band density in the contralateral kidney of sham rats, UUO rats, EPL rats, and HJHR rats. Data represent the mean ± SEM (*n* = 3). ^*∗*^*P* < 0.05 vs. the sham group; ^#^*P* < 0.05 vs. the UUO group.

**Figure 6:**
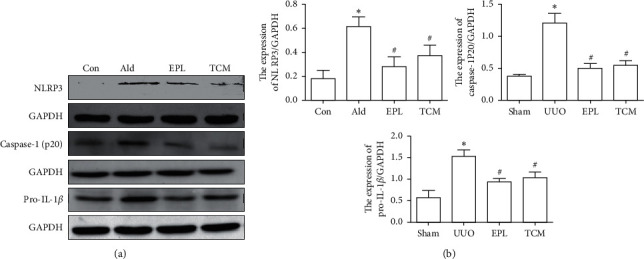
Inhibitory effect of HJHR on inflammation in the mesangial cells. (a) Western blot analysis of NLRP3, pro-caspase-1, pro-IL-1*β*, caspase-1-p20, and mature-IL-1*β* in the mesangial cells. (b) Quantification of NLRP3/GAPDH, pro-caspase-1/GAPDH, caspase-1-p20/GAPDH, pro-IL-1*β*/GAPDH, and mature-IL-1*β*/GAPDH protein band density in the contralateral kidney of sham, UUO, and EPL rats. Data represent the mean ± SEM (*n* = 3). ^*∗*^*P* < 0.05 vs. the sham group; ^#^*P* < 0.05 vs. the UUO group.

Pyroptosis is accompanied by caspase-1 activation and secretion of the proinflammatory factor IL-1*β*. Activation of caspase-1 leads to the formation of membrane pores, which in turn leads to cellular lysis and leakage of the cytosolic contents. Aldo-induced NLRP3 activation results in caspase-1 cleavage and activation. As shown in Figures 5 and 6, caspase-1 and mature-IL-1*β* protein were significantly increased in CK of UUO compared with rats in the sham group. Importantly, mature-IL-1*β* and caspase-1 protein were inhibited in the HJHR group and eplerenone group.

### 3.6. The Expression of Serum- and Glucocorticoid-Inducible Protein Kinase-1 (SGK-1) and Nuclear Factor *κB* (p65) Decreased in the Contralateral Kidney by HJHR and Eplerenone

**Figure 7:**
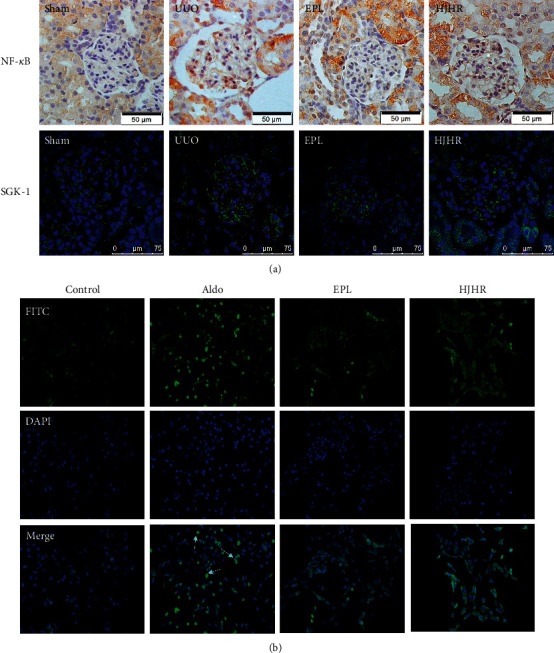
The expression of SGK-1/NF-*κB*. (a) Expression of SGK-1 and NF-*κB* is shown weakly in rats of the sham group. In UUO rats, it was markedly increased in glomeruli. (b) HJHR reduced NF-*κB* activation in Aldo-induced mesangial cells.

There are experimental data which support the hypothesis that serum aldosterone is increased in the acute UUO model [9], which was confirmed in our study (Figure 7). It was reported that aldosterone contributes to the progression of mesangial cell injury [10]. To determine the signalling pathway of Aldo/SGK-1/NF-*κB* in relation to inflammation in mesangial cells, we assessed the activation of NF-*κB* in vivo and vitro.  Figure 7(a) shows the expression of SGK-1 in CK of the four groups. As compared with the control group, the expression of in the UUO group was significantly increased. In the HJHR group, the expression of SGK-1was downregulated.  Figure 7(b) shows that HJHR reduced NF-*κB* activation in aldosterone-induced mesangial cells.

## 4. Discussion

Obstructive nephropathy is a common entity that occurs at all, which is usually unilateral. Loss of function in the CK has attracted significant consideration for its role in the progression of chronic renal failure. Our previous study found that cell pyroptosis in contralateral kidneys of rat with UUO is associated with the MR/SGK-1 pathway [3]. However, the exact mechanisms responsible for the contralateral glomerular inflammation induced by UUO remain unclear. In this study, we used the UUO model to explore the mechanisms of the mesangial cell pyroptosis of CK in the progression of chronic renal failure.

In this study, renal cortical sections of UUO rats showed greater numbers of TUNEL-positive MCs. Because inhibition of the MR-blocker in in vivo and in vitro studies was associated with reduction in the number of TUNEL-positive cells, it appears that eplerenone ameliorates cell pyroptosis which is mediated through the MR pathway. Macrophages are rare in the healthy renal cortex [11]; however, 10 days after UUO, a large number of macrophages accumulate in the contralateral glomerulus. We also found that the expression of NLRP3, caspase-1, and IL-1*β* is closely related with mesangial cell damage of contralateral glomerular after UUO, whereas MR antagonist eplerenone reversed the enhancement. Eplerenone significantly inhibited the inflammatory cell infiltration and cell pyroptosis in the contralateral glomerulus of UUO rats. These results indicate that eplerenone inhibits the progression of glomerular inflammation.

In the kidney, MR has been localised to mesangial cells, preglomerular vasculature, and fibroblasts as well as distal tubular cells of the nephron [12]. A growing body of experimental and clinical evidence has accumulated to support the contribution of MR to renal damage. More recent experiments showed that the activation of MR of mesangial cells within the glomerulus appears to lead to glomerular sclerosis [13]. Akira Nishiyama [8] indicates that MR is expressed in cultured rat mesangial cells and involved in aldosterone-induced rat mesangial cell injury. Our study indicates that the mesangial cell is a target for injuries induced by aldosterone. Mesangial cells are local modulators of innate and adaptive immune responses. In addition, mesangial cells can secrete a wide range of proinflammatory mediators in response to injury [14, 15]. Persistence of infiltrating macrophages makes mesangial cells release many growth factor and inflammatory mediators involved in cell proliferation and matrix deposition, which results in the development of glomerulosclerosis and decreased glomerular filtration rate [16].

Inflammation plays a key role in the onset and progression of renal injury after UUO [2]. Glomerular inflammation is the major process involved in the progression of glomerulosclerosis. Here, we analysed the processing of caspase-1 and IL-1*β* after UUO in rats, which suggested activation of the NLRP3 inflammasome during CK injury. Compared with the sham group, UUO rats had more mesangial cell injury, inflammation, and fibrosis associated with the enhancement in caspase-1 activation and maturation of IL-1*β*. These data confirm that the NLRP3 inflammasome upregulates these cytokines in the CK during injury.

An inflammatory response is induced during cellular injury, such as necrosis and pyroptosis. Pyroptosis is a proinflammatory form of regulated cell death that is triggered by inflammatory caspase-1. Cellular contents that are inappropriately released after loss of plasma membrane integrity are endogenous adjuvants or danger-associated molecular patterns (DAMPs) [17–19]. Upon DAMP stimulations, NLRP3 molecules recruit adaptor proteins to form a large platform for pro-caspase-1 binding, leading to its activation by autocatalytic processing. The active caspase-1 in turn proteolytically cleaves 31kD pro-IL-1*β* into 17kD mature-IL-1*β*, which is subsequently released to potentiate the innate immunity or inflammation. Concomitantly, active caspase-1 also cleaves gasdermin D (GSDMD) to produce an N-terminal fragment (GSDMD-NT), which forms pores on the plasma membrane, thereby mediating the programmed cell death known as pyroptosis [20]. Some studies have indicated that cell membrane rupture and pyroptosis is required for the release of IL-1*β* and other inflammatory factors [21], suggesting that pyroptosis is an important process in mediating inflammation.

Previous studies have shown that UUO immediately activates the renin-angiotensin system (RAS), increasing renin, angiotensin II (AII), and aldosterone serum levels and inflammatory cells infiltrated [7, 22]. We hypothesised that UUO could increase aldosterone via activating RAS and then affect CK through blood circulation. This was confirmed by in vitro experiments. In vitro, we investigated the expression of MR in aldosterone-treated MCs using immunofluorescence methods. Immunofluorescence analysis with MR-specific antibody detected MR is predominantly present in the cytoplasm of MCs. This experiment showed that MR translocation from the cytoplasm to the nucleus was induced in MCs by treatment with aldosterone, and the cellular MR translocation was prevented by eplerenone. Furthermore, we found that aldosterone not only promoted MR activation but also contributed to NLRP3 inflammasome activation which are important in the progression of glomerular injury. The NLRP3 inflammasome, which is related to kidney diseases, cardiovascular diseases, human immunodeficiency virus (HIV) infection, and Alzheimer's disease, is the most studied type [23]. Previous studies have demonstrated that NLRP3-deficient mice had less damage regarding inflammation and fibrosis after UUO, as well as less activation of caspase-1 and release of mature-IL-1*β* [6]. Hua KF [24] confirms that ROS generation and activation of NF-*κB* and the NLRP3 inflammasome are crucial mechanistic events involved in the progression of the renal disorder. Simiao pill suppressed NF-*κB*/NLRP3 inflammasome activation to reduce IL-1*β* in renal glomeruli of high fructose-fed rats [25]. Wuling San suppressed NLRP3 inflammasome activation to reduce IL-1*β* production in high fructose-induced hyperuricemic mice [26]. Dae Gill Kang [27] convinced that the retention of water and salt in glycyrrhizin-treated rats is, at least in part, causally related to the MR. Our findings indicated that MR antagonists and HJHR may serve as useful therapeutic targets for the treatment of glomerular inflammatory disease. The pathogenesis of chronic kidney disease is the origin deficiency and symptom evil, tonifying the kidney and strengthening the spleen is the common method. The symptom evil is related to the five internal organs, and pathological products such as water-wet, turbidity, and blood stasis are common in symptom evil, commonly used dampness, turbidity, blood circulation, etc. Toxin damaging kidney collaterals run through the whole process of chronic kidney disease, and method of Huoxue Jiedu Huayu is the basic principle of TCM in the treatment. In this prescription, Dilong, Biejia, and Chishao were used to remove blood stasis, Huangqin and Jinyinhua were used to attack the toxic, and Huangqi was used to recover healthy qi. This has also been confirmed by the results of modern pharmacological research [28]. We attained satisfied therapeutic effects of HJHR on the treatment of kidney disease in our previous clinical practice. In the experiment, it was observed that HJHR can inhibit the mesangial cell pyroptosis, which may be related to downregulation of the expression of NLRP3 in renal tissue. Nevertheless, further exploration on the deeper mechanism is needed.

## 5. Conclusion

The present study provides evidence, for the first time, that UUO induced MC pyroptosis in CK of UUO rats. Aldosterone plays an important role in inflammation of the CK in the UUO model through cell pyroptosis via MR/NLRP3-capase1-IL-1*β* pathways. Our studies suggest that MR may serve as a potential target for survival of renal injury, such as obstructive nephropathy. Further studies are currently under way to elucidate the precise molecular mechanisms by which UUO mediates glomerular cell injury via activation of MR. In addition, determining how MR expression is regulated during the development of glomerular injury may lead to a better understanding of the pathophysiology of UUO-induced renal injury.

## Data Availability

The data used to support the findings of this study are available from the corresponding author upon request.

## References

[1] Wang F., Yang C., Long J. (2019). Executive summary for the 2015 annual data report of the China kidney disease network (ck-net). *Kidney International*.

[2] Bai K., Jeremiah M. (2002). Obstructive nephropathy and renal fibrosis. *American Journal of Physiology-Renal Physiology*.

[3] Waasdorp M., De Rooij D. M., Florquin S., Duitman J., Spek C. A. (2019). Protease-activated receptor-1 contributes to renal injury and interstitial fibrosis during chronic obstructive nephropathy. *Journal of Cellular and Molecular Medicine*.

[4] Meng X.-M., Nikolic-Paterson D. J., Lan H. Y. (2014). Inflammatory processes in renal fibrosis. *Nature Reviews Nephrology*.

[5] Lee S. B., Kalluri R. (2010). Mechanistic connection between inflammation and fibrosis. *Kidney International*.

[6] Vilaysane A., Chun J., Seamone M. E. (2010). The NLRP3 inflammasome promotes renal inflammation and contributes to CKD. *Journal of the American Society of Nephrology*.

[7] Li D. T., Gaber L., Eknoyan G. (2011). Obstructive uropathy. *Contributions to Nephrology*.

[8] Nishiyama A., Yao L., Fan Y. (2005). Involvement of aldosterone and mineralocorticoid receptors in rat mesangial cell proliferation and deformability. *Hypertension*.

[9] Nagai J.-L., Schanstra J. P. (2005). Obstructive nephropathy: insights from genetically engineered animals. *Kidney International*.

[10] Terada Y., Ueda S., Hamada K. (2012). Aldosterone stimulates nuclear factor-kappa *B* activity and transcription of intercellular adhesion molecule-1 and connective tissue growth factor in rat mesangial cells via serum-and glucocorticoid-inducible protein kinase-1. *Clinical and Experimental Nephrology*.

[11] Taniguchi G. F., Unanue E. R. (1984). Origin of the rat mesangial phagocyte and its expression of the leukocyte common antigen. *Laboratory Investigation; a Journal of Technical Methods and Pathology*.

[12] Remuzzi G., Cattaneo D., Perico N. (2008). The aggravating mechanisms of aldosterone on kidney fibrosis. *Journal of the American Society of Nephrology*.

[13] Brem A. S., Gong R. (2015). Therapeutic targeting of aldosterone: a novel approach to the treatment of glomerular disease. *Clinical Science*.

[14] Gomez-Guerrero C., Hernandez-Vargas P., Lopez-Franco O., Ortiz-Munoz G., Egido J. (2005). Mesangial cells and glomerular inflammation: from the pathogenesis to novel therapeutic approaches. *Current Drug Target-Inflammation & Allergy*.

[15] Schlöndorff D., Banas B. (2009). The mesangial cell revisited: no cell is an Island. *Journal of the American Society of Nephrology*.

[16] Klahr S., Schreiner G., Ichikawa I. (1988). Progression of renal disease. *New England Journal of Medicine*.

[17] Shi Y., Zheng W., Rock K. L. (2000). Cell injury releases endogenous adjuvants that stimulate cytotoxic T cell responses. *Proceedings of the National Academy of Sciences*.

[18] Ishii K. J., Suzuki K., Coban C. (2001). Genomic DNA released by dying cells induces the maturation of APCs. *The Journal of Immunology*.

[19] Kohn P., Capobianco A., Scaffidi P. (2004). HMGB1 is an endogenous immune adjuvant released by necrotic cells. *EMBO Reports*.

[20] Shi J., Zhao Y., Wang K. (2015). Cleavage of GSDMD by inflammatory caspases determines pyroptotic cell death. *Nature*.

[21] Zhuang W.-T., Wan H., Hu L. (2015). Gasdermin D is an executor of pyroptosis and required for interleukin-1*β* secretion. *Cell Research*.

[22] Yang M., Corradetti V., Rocca C. (2016). Mesenchymal stromal cells prevent renal fibrosis in a rat model of unilateral ureteral obstruction by suppressing the renin-angiotensin system via HuR. *PLoS One*.

[23] Iwata M., Ota K. T., Duman R. S. (2013). The inflammasome: pathways linking psychological stress, depression, and systemic illnesses. *Brain, Behavior, and Immunity*.

[24] Hua K.-F., Yang S.-M., Kao T.-Y. (2013). Osthole mitigates progressive IgA nephropathy by inhibiting reactive oxygen species generation and NF-*κB*/NLRP3 pathway. *PLoS One*.

[25] Ma C.-H., Kang L.-L., Ren H.-M., Zhang D.-M., Kong L.-D. (2015). Simiao pill ameliorates renal glomerular injury via increasing Sirt1 expression and suppressing NF-*κB*/NLRP3 inflammasome activation in high fructose-fed rats. *Journal of Ethnopharmacology*.

[26] Yang Y., Zhang D.-M., Liu J.-H. (2015). Wuling San protects kidney dysfunction by inhibiting renal TLR4/MyD88 signaling and NLRP3 inflammasome activation in high fructose-induced hyperuricemic mice. *Journal of Ethnopharmacology*.

[27] Ding D. G., Sohn E. J., Lee H. S. (2003). Effects of glycyrrhizin on renal functions in association with the regulation of water channels. *The American Journal of Chinese Medicine*.

[28] Zhang Y.-W., Wu C.-Y., Cheng J.-T. (2007). Merit of Astragalus polysaccharide in the improvement of early diabetic nephropathy with an effect on mRNA expressions of NF-*κB* and *IκB* in renal cortex of streptozotoxin-induced diabetic rats. *Journal of Ethnopharmacology*.

